# Supplementary material for: Catchments catch all in South African coastal lowlands: topography and palaeoclimate restricted gene flow in Nymania capensis (Meliaceae)—a multilocus phylogeographic and distribution modelling approach
Source: PeerJ. 2017 Jan 31;5:e2965. doi: 10.7717/peerj.2965 (PMC5289106; doi:10.7717/peerj.2965)
Supplement: Supplemental Information 1 — Appendices S1, S2 and S3. [file peerj-05-2965-s001.docx]

**Appendix S1** The growth habit (A), flowers (B), and inflated seed capsule (C) of *Nymania capensis* (Meliaceae). The inflated capsule is peculiar in this species as it is not known anywhere else in the Meliaceae ([Pennington & Styles, 1975](#_ENREF_2)).


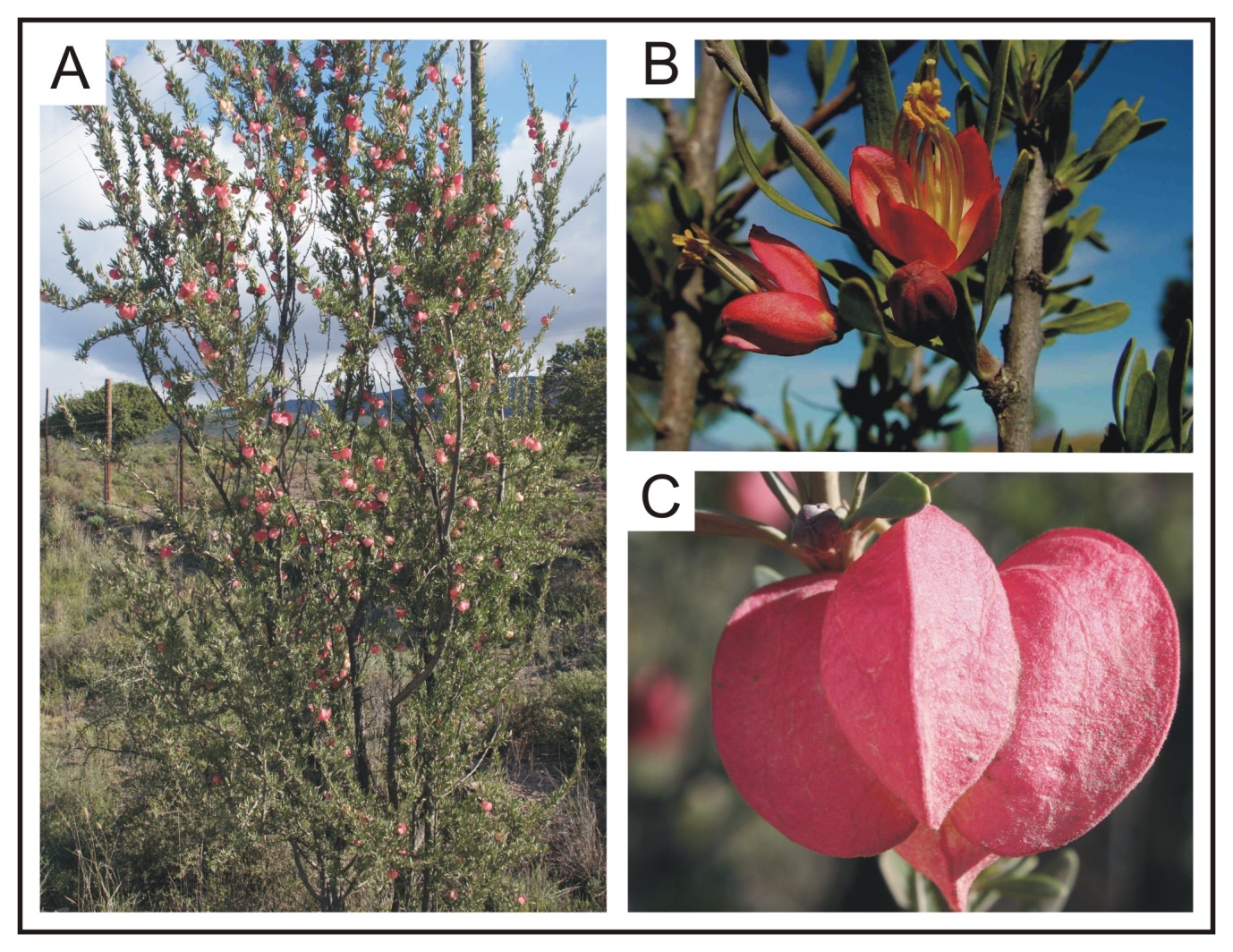


**Appendix S2** Additional methodological details.

## Samples and DNA data

Extraction protocols and PCR protocols for the ITS sequences follow those described in Potts et al. (2014); of note is that high fidelity Taq polymerase was used (Kapa HiFi Taq; Kapa Biosystems, Boston, Massachusetts, United States). For the ncpGS region, the PCRs were performed in volumes of 36 μl containing 1.2 μl of template DNA, 3.6 μl of 10X Kapa Taq polymerase reaction buffer (Kapa Biosystems), 0.72 μl MgCl2 (50mM), 1.2 μl of each primer (10 μM), 1.44 μl of dNTPs (10 mM), 0.24 μl Taq polymerase and sterile H2O up to 36 μl. The PCR protocol consisted of an initial 5 min denaturing step at 95°C; 30 cycles, each comprising 95°C for 1 min, 50°C for 1 min, 72°C for 2 min; and a final 7 min extension step at 72°C. All sequencing was performed by either Macrogen (Seoul, Korea) or the University of Stellenbosch Sequencing Facility (Cape Town, South Africa).

**Phylogenetic and phylogeographic analyses**

The level of genealogical divergence was assessed using the genealogical sorting index (gsi, Cummings et al., 2008). The gsi statistic is a standardised measure of the extent to which predefined groups in a gene tree exhibit exclusive ancestry (fall within a distinct clade); the gsi statistic ranges from 0 (a complete lack of genealogical divergence with respect to other groups) to 1 (monophyly, all elements of a predefined group fall within the same, exclusive subtree). The significance of the gsi statistic is obtained through randomisation of the group labels across the tips in a gene tree. Thus, lineages within groups can be tested against a null hypothesis of no divergence. The gsi was calculated on 100 topologies sampled from the 1000 maximum likelihood bootstrap replicates (generated using RAxML) for each DNA region. These 100 individual gsi measurements were then used to calculate an ensemble gsi statistic gsiT for each DNA region. The gsiT statistic represents a summary of genealogical exclusivity that incorporates tree topology uncertainty. All analyses were performed using the GENEALOGICALSORTING library version 0.91 (available from http://www.genealogicalsorting.org/) implemented in R 2.15.

Note that analyses of molecular variance would be a suitable statistical framework to assess the level of genealogical divergence; however, all software at present cannot accommodate 2ISPs in the DNA alignment (such sites are usually removed prior to analysis; e.g. Arlequin version 3.01 - Excoffier et al., 2005). Thus, analyses of genealogical divergence were restricted to the genealogical sorting index that, as a tree-based index, is unaffected by the presence of 2ISPs in the DNA alignment.

**Molecular dating**

The dating of lineage divergence was carried out using Beast version 1.4.8 ([Drummond & Rambaut, 2007](#_ENREF_1)), which estimates the tree topology and the date (height) of nodes simultaneously using a Bayesian approach. The Beast analysis was performed using all cpDNA samples as this sampling is important for coalescent estimation of lineage divergence. The chloroplast dataset were analysed using the K3Puf substitution model, as selected by the Akaike Information Criterion in jModelTest version 0.1.1 ([Posada, 2008](#_ENREF_3)), and the Dollo model for indels. The hypothesis of rate constancy between samples was rejected using the relative rate test ([Tajima, 1993](#_ENREF_4)) as implemented within the ape library in R using an outgroup sample (BOL48535). Therefore, an uncorrelated lognormal clock was used for all Beast analyses. All other priors were left to the defaults. The Beast analysis were replicated (N = 3) to verify convergence to stationarity and the estimation of effective sample size (ESS); this was confirmed by visual inspection of each analysis. Each analysis was run for 10^7^ generations, sampling every 10^4^ generations (i.e. a total sampling of 1000 trees). After discarding the first 200 samples as burnin, the parameter and tree estimates from the three runs were combined to yield a consensus of maximum clade credibility (posterior probability limit set to 0.5) and median and 95% posterior densities (HPD) of age estimates. Molecular dating using nDNA alignments was not possible, as intra-individual variant variability was observed and variant sequences of known phase were unavailable for most samples.

## 2ISP detection: Direct-PCR versus cloning

Tests of how successful direct-PCR sequencing is for detecting 2ISPs are limited. To assess this, eight samples were cloned and eight clones were sequenced per sample. The 2ISPs detected in direct-PCR ITS sequences were compared with the variable sites found in the clones for each individual. Cloning for this comparison was performed using the pGEM-T Easy Vector System II (Promega Wisconsin, USA) following the manufacturer’s instructions, but downscaled to half reactions. To facilitate cloning, Kapa HiFi PCR products were incubated at 72°C for 10 minutes with Kapa Taq polymerase to provide 5´ terminal thymidine overhangs. These results were then compared with two similar studies that compared direct-PCR and cloned sequences ([Rosselló *et al.*, 2007](#_ENREF_52); [Yamaji *et al.*, 2007](#_ENREF_72)).

Direct-sequencing was found to detect the majority (77%) of 2ISPs in *Nymania capensis* when compared to the variable sites observed in the clones (Table S2.1). There is also a low percentage of false-positives (5.5%; Table S2.1) in direct-sequencing alignment (i.e. 2ISPs not observed in the clone sample); false positives might arise from insufficient sampling of the intra-individual clone population rather than false 2ISP detection in the direct sequencing. These results are similar to those reported by Rosselló *et al.* ([2007](#_ENREF_51)) and Yamaji *et al.* ([2007](#_ENREF_71)) (Table S2.1).

**Table S2.1** Comparison of intra-individual site polymorphisms detected from direct-PCR sequencing versus cloning across multiple sequences from three plant taxa. The number of samples and number of clones per sample are shown in brackets.

|  | Direct sequences | | | | | | | |
| --- | --- | --- | --- | --- | --- | --- | --- | --- |
|  | *Nymania capensis*  (8;8) | |  | *Buxus balearica*^1^  (5; 3-11) | |  | *Asarum sect. Asiasarum*^2^  (30; 13-31) | |
| Clone sequences | Present | Absent |  | Present | Absent |  | Present | Absent |
| Present | 69 | 20 |  | 29 | 4 |  | 177 | 54 |
| Absent | 4 | - |  | 3 | - |  | 1 | - |

^1^ Rossello et al. (2007); ^2^ Yamaji et al. (2007)

**Appendix S3** Additional results: DNA alignments and networks.

**Table S3.1** Variable sites across the chloroplast DNA haplotypes from two gene regions (trnQ-5´–rps16 and atpI–atpH) of *Nymania capensis* accessions. All sequences are compared to the reference haplotype A. The relationships between haplotypes is shown above in Fig. S2.


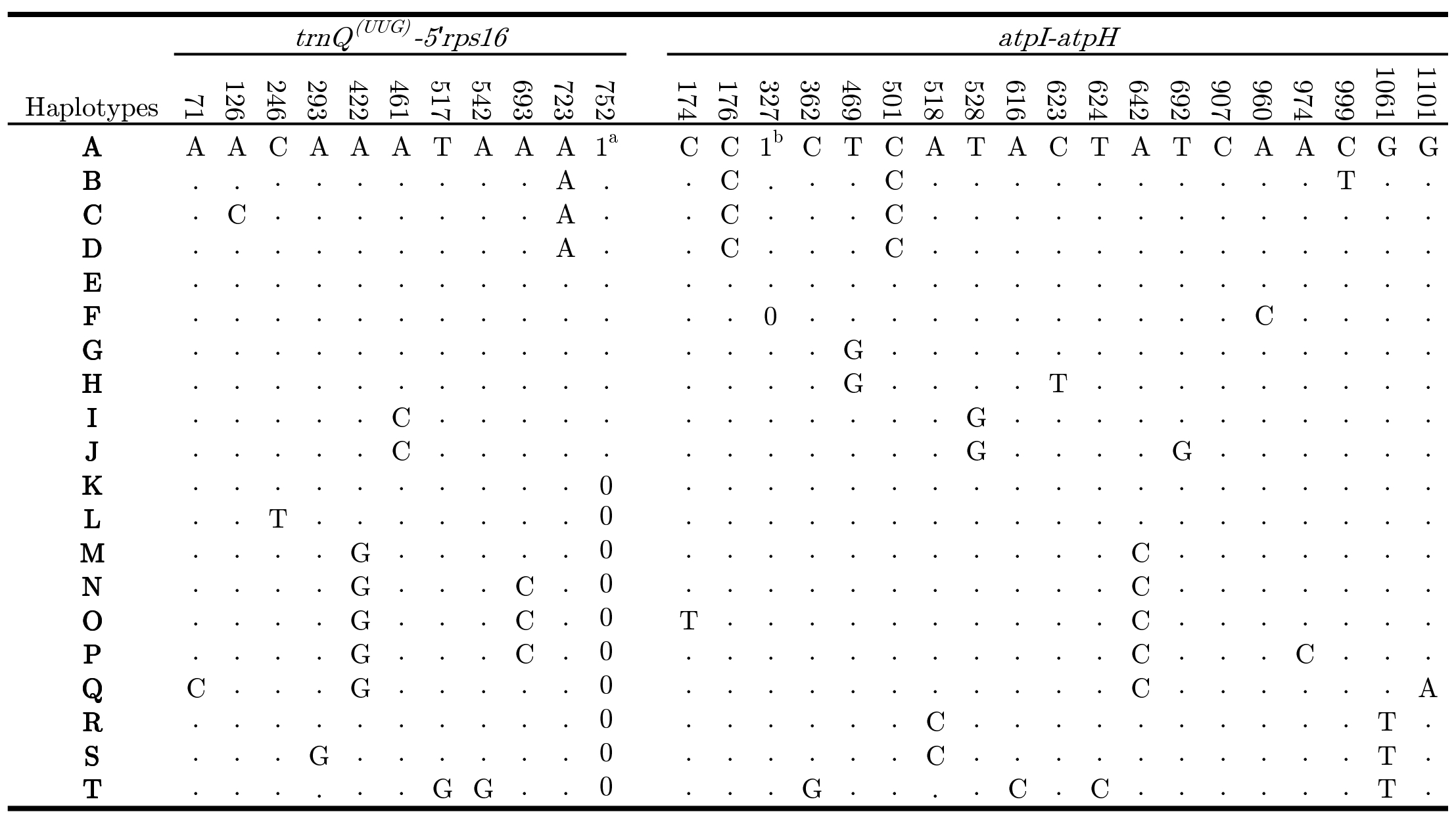


Number ‘0/1’ in the sequences indicate the absence/presence of length polymorphisms whereby the superscripts identify corresponding character states. Note that poly-T stretches were excluded from analyses.

a, TAAGA; b, AAT

**Table S3.2** Variable sites in direct-PCR ncpGS sequences from *Nymania capensis* accessions. All sequences are compared to the consensus sequence. Sequence polymorphisms follow IUPAC codes. Nucleotide positions refer to the aligned sequences in the dataset.


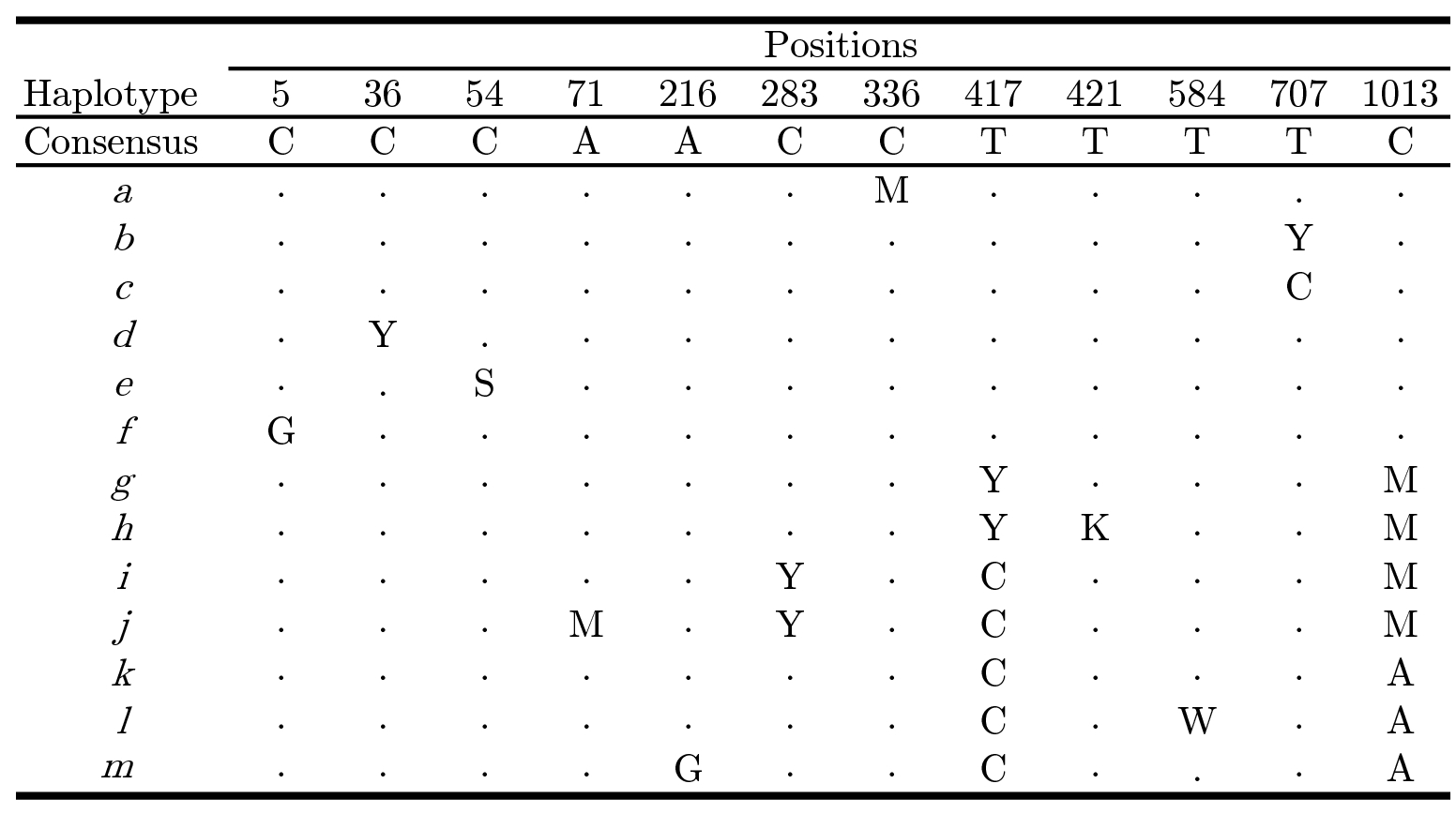


**Table S3.3** Variable sites in direct-PCR ribosomal ITS sequences from a subset of *Nymania capensis* accessions. Five samples were selected at random from each ITS cluster to demonstrate the variability within and between the clusters. All sequences are compared to the reference consensus sequence. Intra-individual site polymorphisms (2ISPs) are coded using IUPAC nomenclature with the exception that 2ISPs involving a base and an indel are coded using ‘X’.


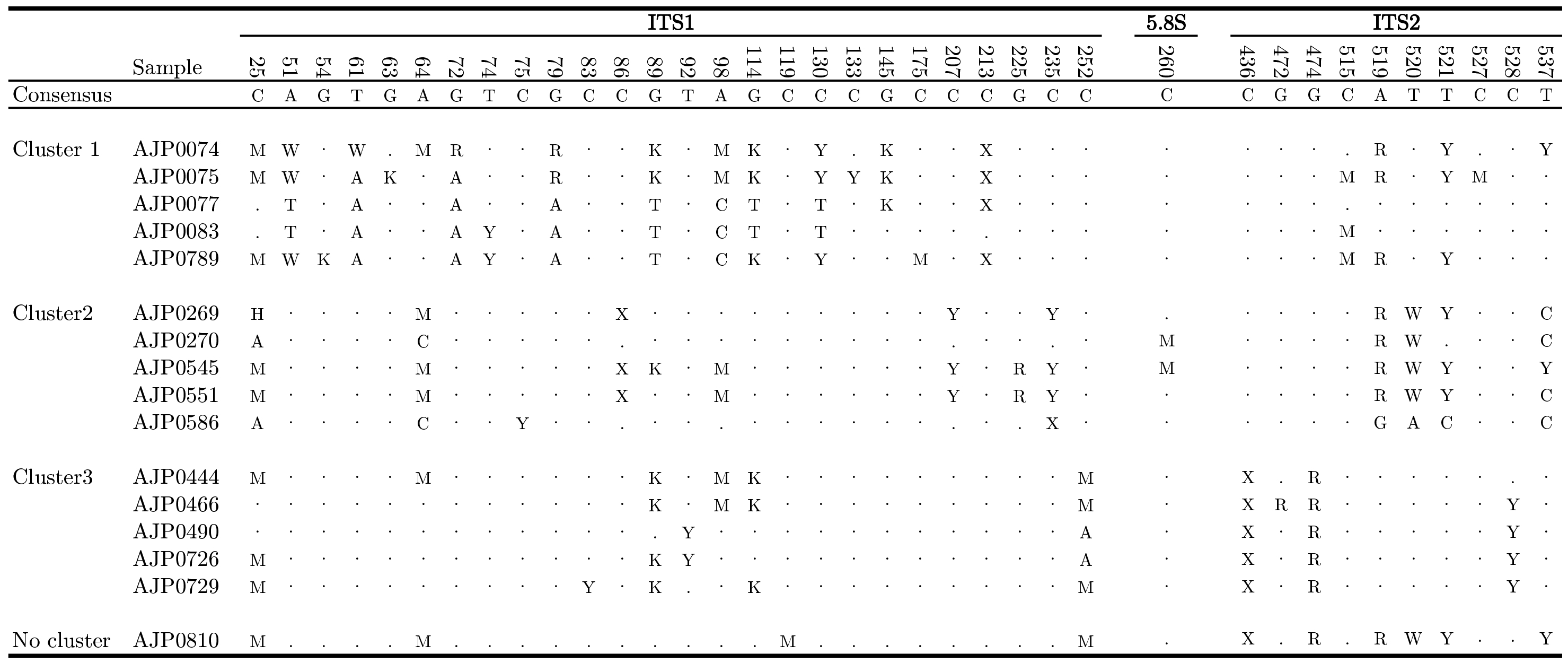


**Table S3.4** Comparison of variable sites between direct-PCR sequences and cloned sequences from eight *Nymania capensis accessions*. Intra-individual individual site polymorphisms are coded using IUPAC nomenclature, with capital letters indicating complete overlap of bases in the trace ﬁle and small letters indicating one dominant base dominant over another in the trace ﬁle. Intra-individual site polymorphisms detected in the direct sequences involving a base and an indel are coded using ‘X’. Clone sequences are compared to the direct sequence from a given accession. A dash (-) denotes a nucleotide indel that may be 1 to 5 bases in length.


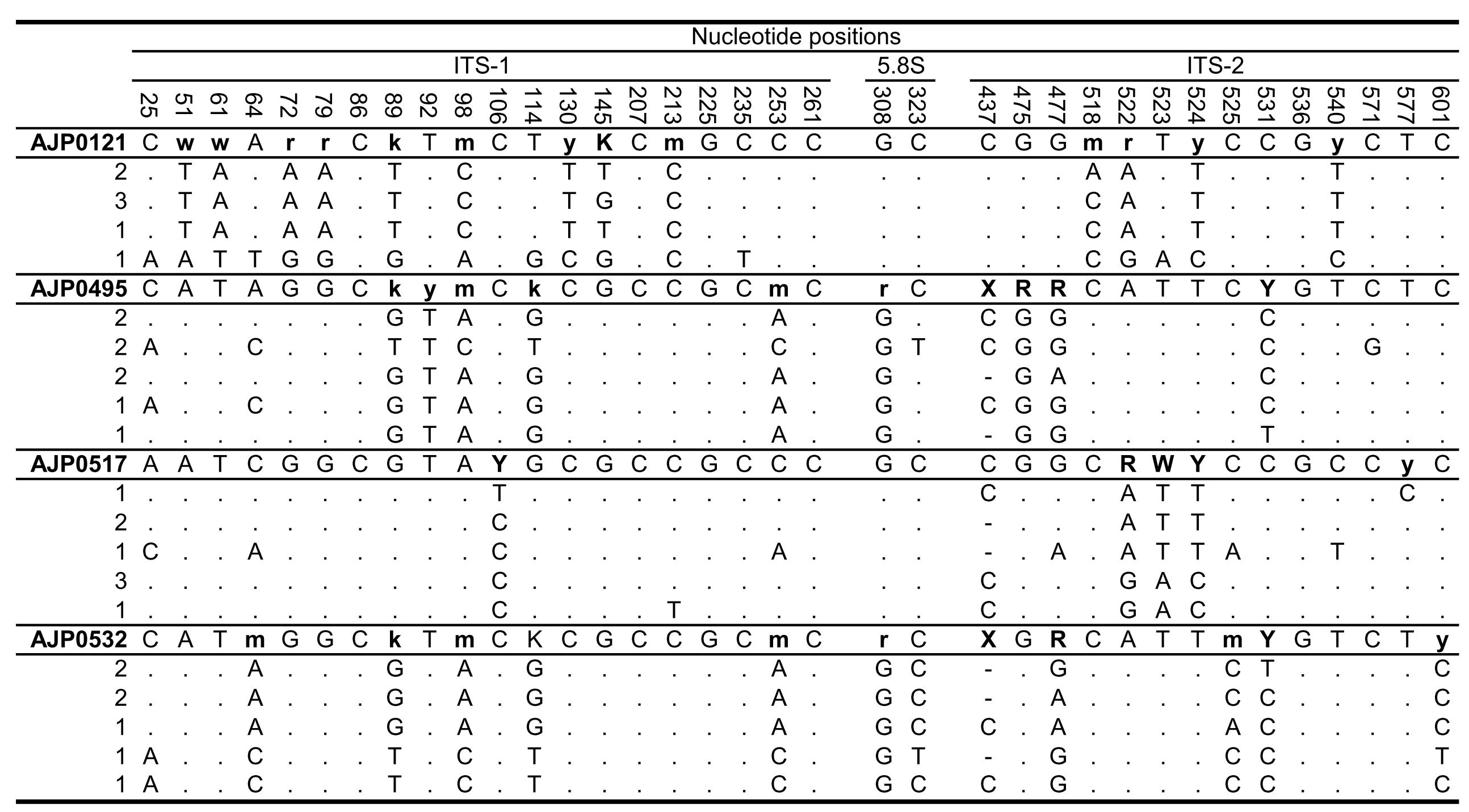


**Table S3.4** continued…


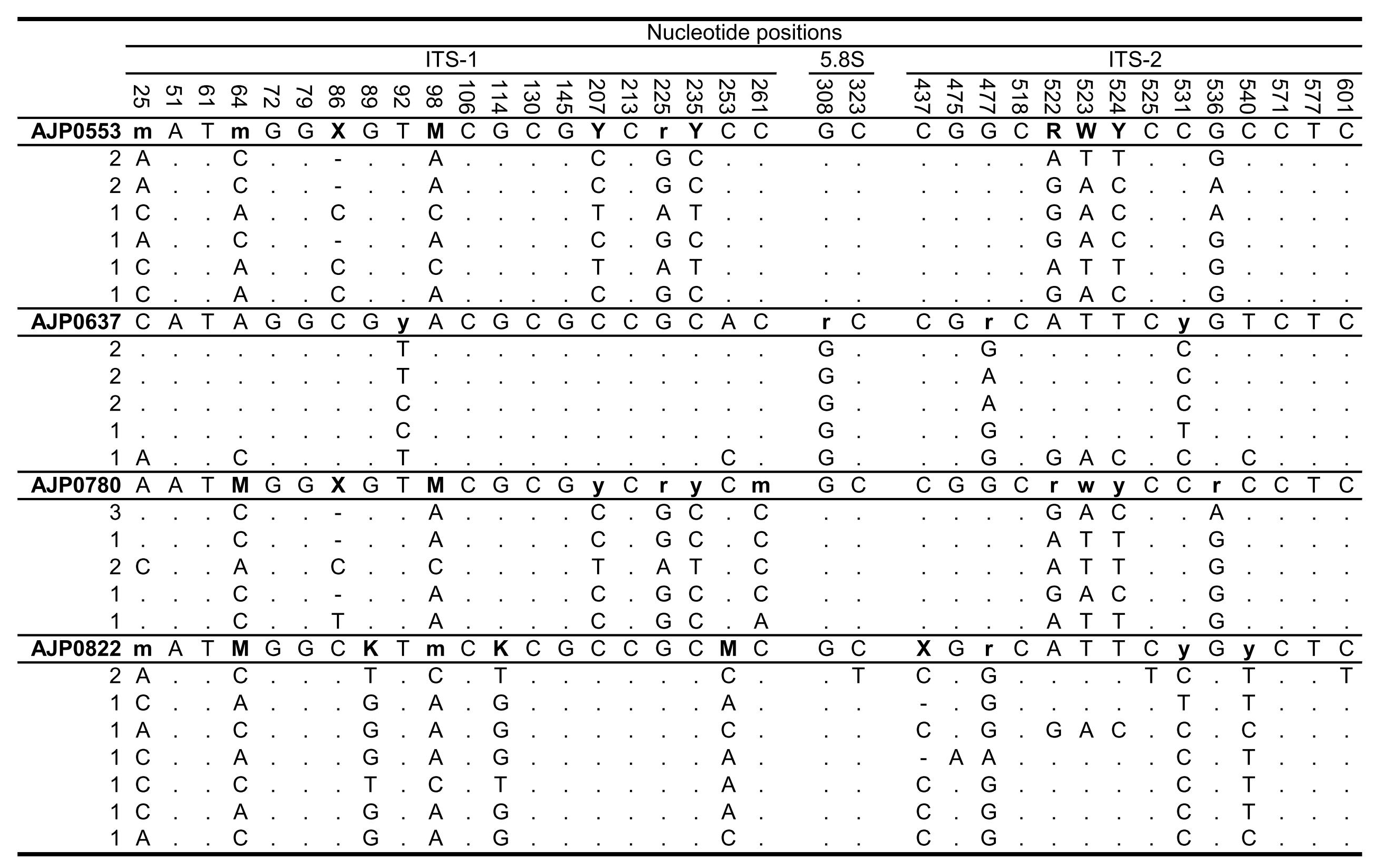


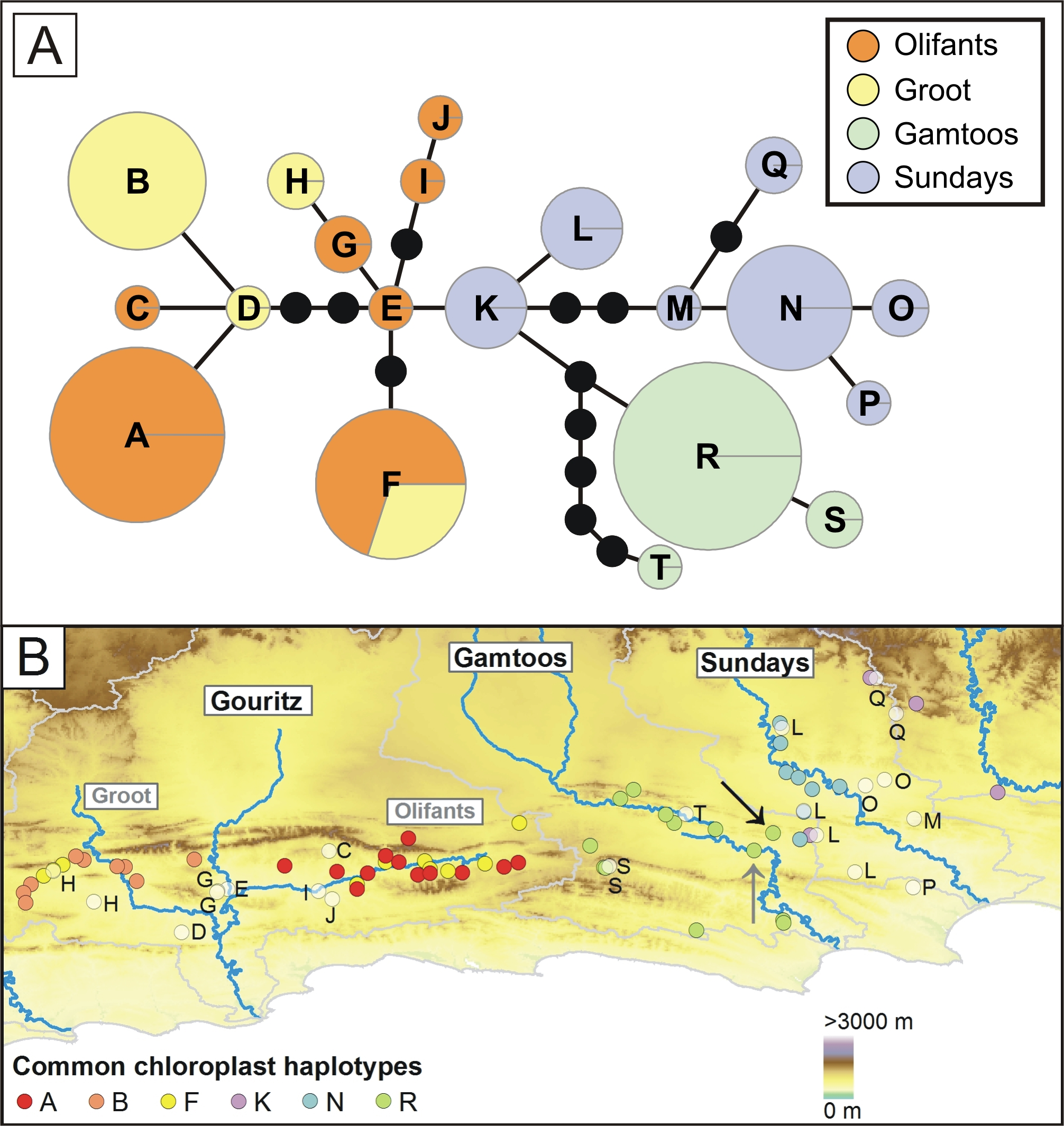


**Figure S3.1** Chloroplast haplotype network and haplotype distribution of *Nymania capensis*. (A) The statistical parsimony haplotype network is based on the two combined chloroplast regions. Lineages are identiﬁed drainage basin are shown. (B) The distribution of lineages within drainage basins (watershed boundaries are shown in grey). Outgroup samples formed an unconnected network. Black and grey arrows represent samples AJP0537 and AJP0810, respectively, which are discussed in the text.


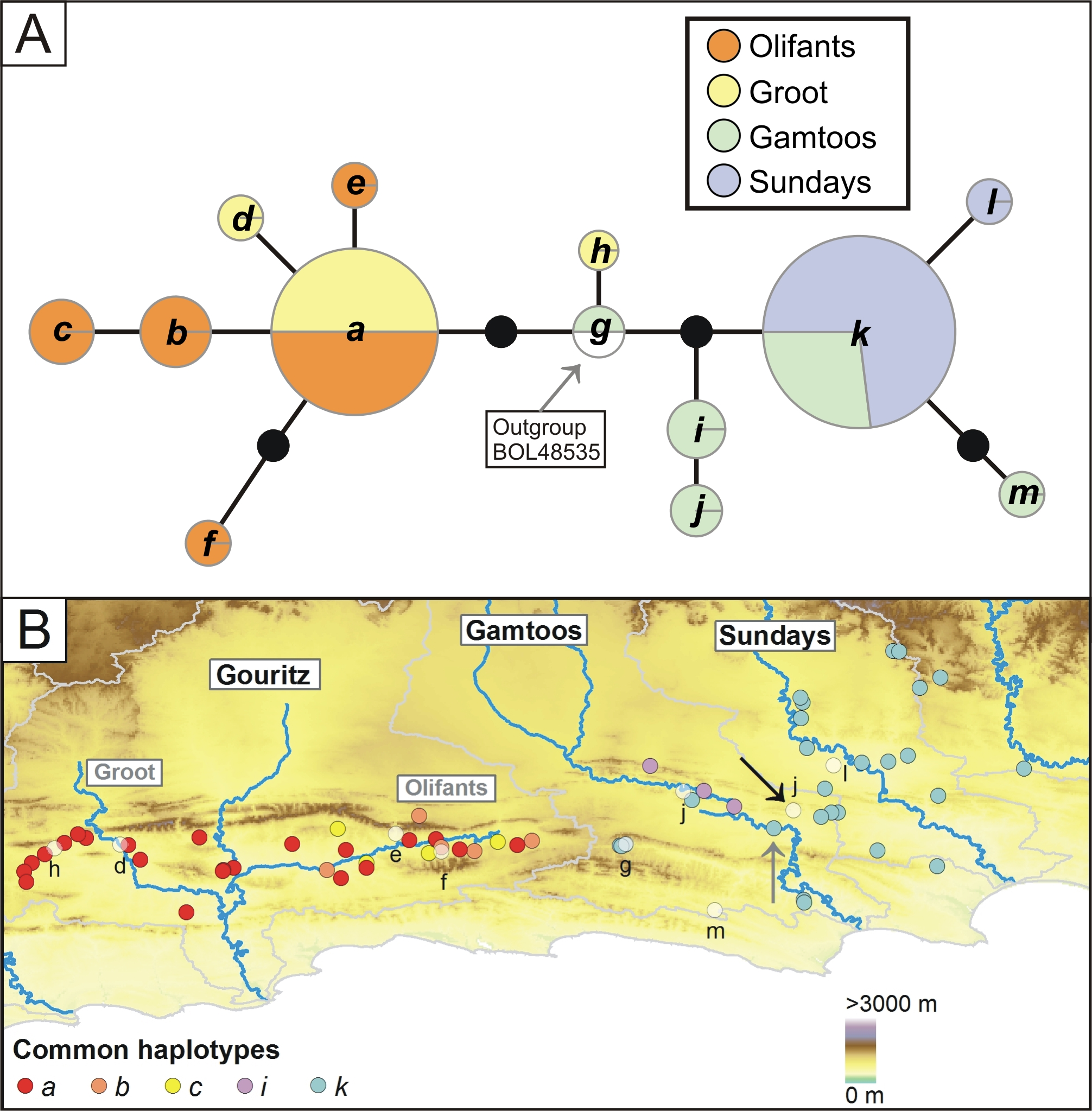


**Figure S3.2** Network, clusters and cluster distribution of ncpGS haplotypes from *Nymania capensis*. (A) The NeighbourNet splits phylogenetic network is based on polymorphism p-distances. The outgroup sample is from the disjunct northern distribution (BOL48535). (B) The distribution of clusters identiﬁed in the network across major drainage basins (watershed boundaries are shown in grey). Black and grey arrows represent samples AJP0537 and AJP0810, respectively, which are discussed in the text.

**References**

Drummond, A.J. & Rambaut, A. (2007) BEAST: Bayesian evolutionary analysis by sampling trees. *BMC Evolutionary Biology*, **7**, 214.

Pennington, T.D. & Styles, B.T. (1975) A generic monograph of the Meliaceae. *Blumea*, **22**, 419-540.

Posada, D. (2008) jModelTest: phylogenetic model averaging. *Molecular Biology and Evolution*, **25**, 1253-1256.

Tajima, F. (1993) Simple methods for testing molecular clock hypothesis. *Genetics*, **135**, 599-607.
