# Supplementary material for: Catchments catch all in South African coastal lowlands: topography and palaeoclimate restricted gene flow in Nymania capensis (Meliaceae)—a multilocus phylogeographic and distribution modelling approach
Source: PeerJ. 2017 Jan 31;5:e2965. doi: 10.7717/peerj.2965 (PMC5289106; doi:10.7717/peerj.2965)
Supplement: Supplemental Information 2 — See the Readme for file descriptions. This zip file includes all DNA alignments. [file peerj-05-2965-s002.zip › SupplementalInformationFiles/DNA.Data/Nymania.capensis_samples.pdf]

Province/Region	District/ Constituency	Voucher Specimen	Latitude	Longitude	Chloroplast hapotype	ITS Cluster	ncpGS haplotype
Eastern Cape	Hankey	AJP0517	-33.806600	24.728710	R	2	*k*
		AJP0518	-33.820320	24.729750	R	2	*k*
	Humansdorp	AJP0586	-33.860020	24.255359	R	2	*m*
	Jansenville	AJP0444	-33.215200	24.839900	N	3	*k*
		AJP0446	-32.755600	24.723500	L	3	*k*
		AJP0466	-33.210417	24.839944	L	3	-
		AJP0490	-32.731370	24.709310	N	3	*k*
		AJP0492	-32.839710	24.713340	N	3	*k*
		AJP0495	-33.000480	24.745880	N	3	*k*
		AJP0498	-33.029240	24.811260	N	3	-
		AJP0628	-33.092010	24.886390	N	3	*l*
		AJP0632	-33.077230	25.035360	N	3	*k*
	Pearston	AJP0726	-32.484020	25.203250	K	3	*k*
		AJP0729	-32.485970	25.234490	Q	3	*k*
	Somerset East	AJP0465	-33.251306	25.442722	M	3	*k*
		AJP0633	-33.070510	25.176080	O	3	*k*
		AJP0637	-33.039110	25.282500	O	3	*k*
		AJP0718	-32.624990	25.455020	K	3	*k*
		AJP0722	-32.679940	25.344410	Q	3	*k*
		AJP0744	-33.107720	25.897590	K	3	*k*
	Steytlerville	AJP0537	-33.329350	24.674060	R	3	*j*
		AJP0540	-33.309960	24.358470	R	2	*i*
		AJP0545	-33.226750	24.198500	T	2	*i*
		AJP0551	-33.276000	24.134070	R	2	*k*
		AJP0553	-33.228690	24.088070	R	2	*j*
		AJP0810	-33.426440	24.568880	R	4	*k*
	Uitenhage	AJP0500	-33.341830	24.909610	L	3	*k*
		AJP0532	-33.342660	24.873420	K	3	*k*
		AJP0533	-33.364260	24.818660	N	3	*k*
		AJP0814	-33.626520	25.436450	P	3	*k*
		AJP0822	-33.542490	25.119780	L	3	*k*
	Willowmore	AJP0259	-33.517931	23.751302	R	-	*k*
		AJP0269	-33.508563	23.782502	S	2	*g*
		AJP0270	-33.513244	23.780121	S	2	*k*
		AJP0273	-33.521486	23.760162	R	2	*k*
		AJP0555	-33.143820	23.841080	R	2	-
		AJP0557	-33.094920	23.912550	R	2	*i*
		AJP0774	-33.275740	23.288660	F	1	-
		AJP0780	-33.399970	23.675520	R	2	-

Province/Region	District/ Constituency	Voucher Specimen	Latitude	Longitude	Chloroplast hapotype	ITS Cluster	ncpGS haplotype
Northern Cape	Namakwaland	BOL48535	-28.256006	17.241669	U	-	*g*
		BOL60966	-28.316668	17.249999	V	-	-
Western Cape	Calitzdorp	AJP0347	-33.634659	21.697334	E	1	*a*
		AJP0358	-33.646765	21.644877	G	1	*a*
		AJP0359	-33.652930	21.639690	G	1	*a*
	George	AJP0312	-33.690933	22.269231	J	1	*a*
	Ladismith	AJP0042	-33.705970	20.968903	H	1	-
		AJP0050	-33.593233	21.201433	B	1	*a*
		AJP0057	-33.474133	21.517933	B	1	*a*
		AJP0083	-33.515213	21.137982	B	1	*a*
		AJP0084	-33.512422	21.097586	B	1	*d*
		AJP0103	-33.478789	20.915313	B	1	*a*
		AJP0113	-33.457254	20.871332	B	1	*a*
		AJP0117	-33.502843	20.800455	F	1	*a*
		AJP0120	-33.531895	20.746276	F	1	*h*
		AJP0121	-33.536824	20.748489	H	1	-
		AJP0129	-33.564117	20.695078	F	1	*a*
		AJP0146	-33.608824	20.626011	B	1	*a*
	Oudtshoorn	AJP0021	-33.509060	22.010440	A	1	*a*
		AJP0065	-33.539567	22.293967	A	1	*a*
		AJP0071	-33.548433	22.463717	A	1	-
		AJP0074	-33.487867	22.561767	F	1	-
		AJP0081	-33.490200	22.631383	A	1	*a*
		AJP0224	-33.610121	22.405158	F	1	*c*
		AJP0229	-33.635754	22.403977	A	1	*a*
		AJP0232	-33.454246	22.560153	A	1	*e*
		AJP0278	-33.428425	22.252120	C	1	*c*
		AJP0319	-33.646264	22.194532	I	1	*b*
	Prince Albert	AJP0789	-33.358260	22.683330	A	1	*b*
	Riversdal	AJP0375	-33.871438	21.447890	D	-	*a*
	Swellendam	AJP0162	-33.657569	20.585944	B	1	*a*
		AJP0280	-33.711470	20.596740	B	1	*a*
	Uniondale	AJP0075	-33.484733	22.774033	F	1	*a*
		AJP0077	-33.527900	22.803783	F	1	*b*
		AJP0080	-33.559400	22.736617	A	1	*c*
		AJP0238	-33.549492	22.802571	A	1	*f*
		AJP0243	-33.491221	23.282956	A	1	*b*
		AJP0274	-33.515851	23.206252	A	1	*a*
		AJP0275	-33.496553	23.102923	F	1	*c*
		AJP0306	-33.548124	22.979498	A	-	*b*
		AJP0307	-33.538869	22.901916	F	1	*a*
